# TBDQ: A Pragmatic Task-Based Method to Data Quality Assessment and Improvement

**DOI:** 10.1371/journal.pone.0154508

**Published:** 2016-05-18

**Authors:** Reza Vaziri, Mehran Mohsenzadeh, Jafar Habibi

**Affiliations:** 1 Department of Computer Engineering, Science and Research Branch, Islamic Azad University, Tehran, Iran; 2 Department of Computer Engineering, Science and Research Branch, Islamic Azad University, Tehran, Iran; 3 Department of Computer Engineering, Sharif University of Technology, Tehran, Iran; University of Waikato (National Institute of Water and Atmospheric Research), NEW ZEALAND

## Abstract

Organizations are increasingly accepting data quality (DQ) as a major key to their success. In order to assess and improve DQ, methods have been devised. Many of these methods attempt to raise DQ by directly manipulating low quality data. Such methods operate reactively and are suitable for organizations with highly developed integrated systems. However, there is a lack of a proactive DQ method for businesses with weak IT infrastructure where data quality is largely affected by tasks that are performed by human agents. This study aims to develop and evaluate a new method for structured data, which is simple and practical so that it can easily be applied to real world situations. The new method detects the potentially risky tasks within a process, and adds new improving tasks to counter them. To achieve continuous improvement, an award system is also developed to help with the better selection of the proposed improving tasks. The task-based DQ method (TBDQ) is most appropriate for small and medium organizations, and simplicity in implementation is one of its most prominent features. TBDQ is case studied in an international trade company. The case study shows that TBDQ is effective in selecting optimal activities for DQ improvement in terms of cost and improvement.

## 1. Introduction

**Fig 1:**
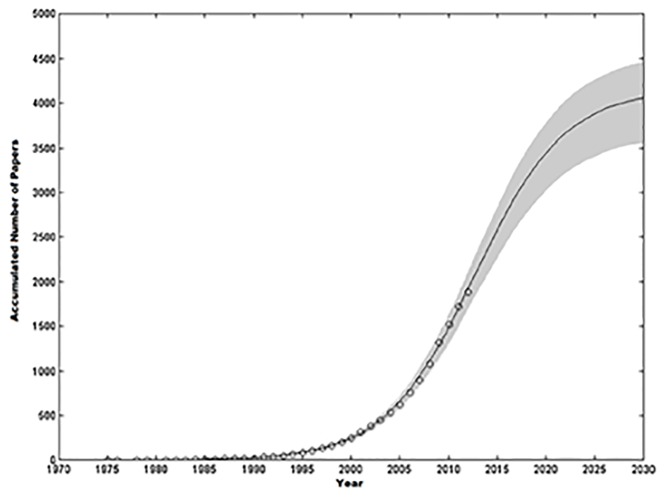
Growth Trend of the DQ Literature [4].

Data quality is often defined as “fitness for use” or data’s ability to meet users’ requirements [1]. Disregard for data quality (DQ) could prove detrimental to business excellence. According to some surveys poor DQ costs U.S. businesses around 600 billion dollars a year [2]. This in turn has encouraged DQ research, which started two decades ago, to enter a new era where a growing number of researchers actively enhance the understanding of data quality problems and develop solutions to the emerging data quality issues[3] (Madnick, Wang, Lee, & Zhu, 2009). The volume of DQ literature since 1970 has increased dramatically with an expected continuous growth till 2030 [4]. This growth is illustrated in Fig 1.

**Fig 2:**
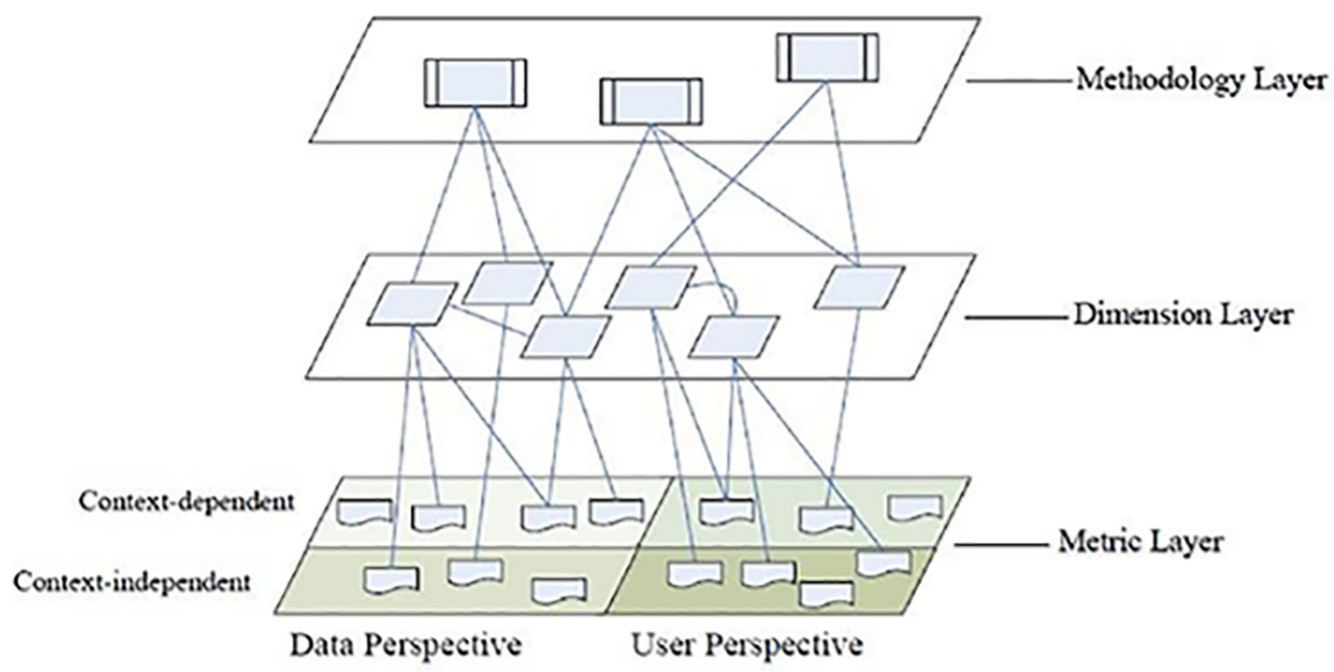
A Framework of DQ Assessment[6].

In order to address DQ issues, DQ methods are designed. A DQ method is a set of guidelines and techniques to define a process for DQ assessment and improvement. [5]. In [6]a layered framework is presented for DQ assessment which is illustrated in Fig 2.

**Fig 3:**
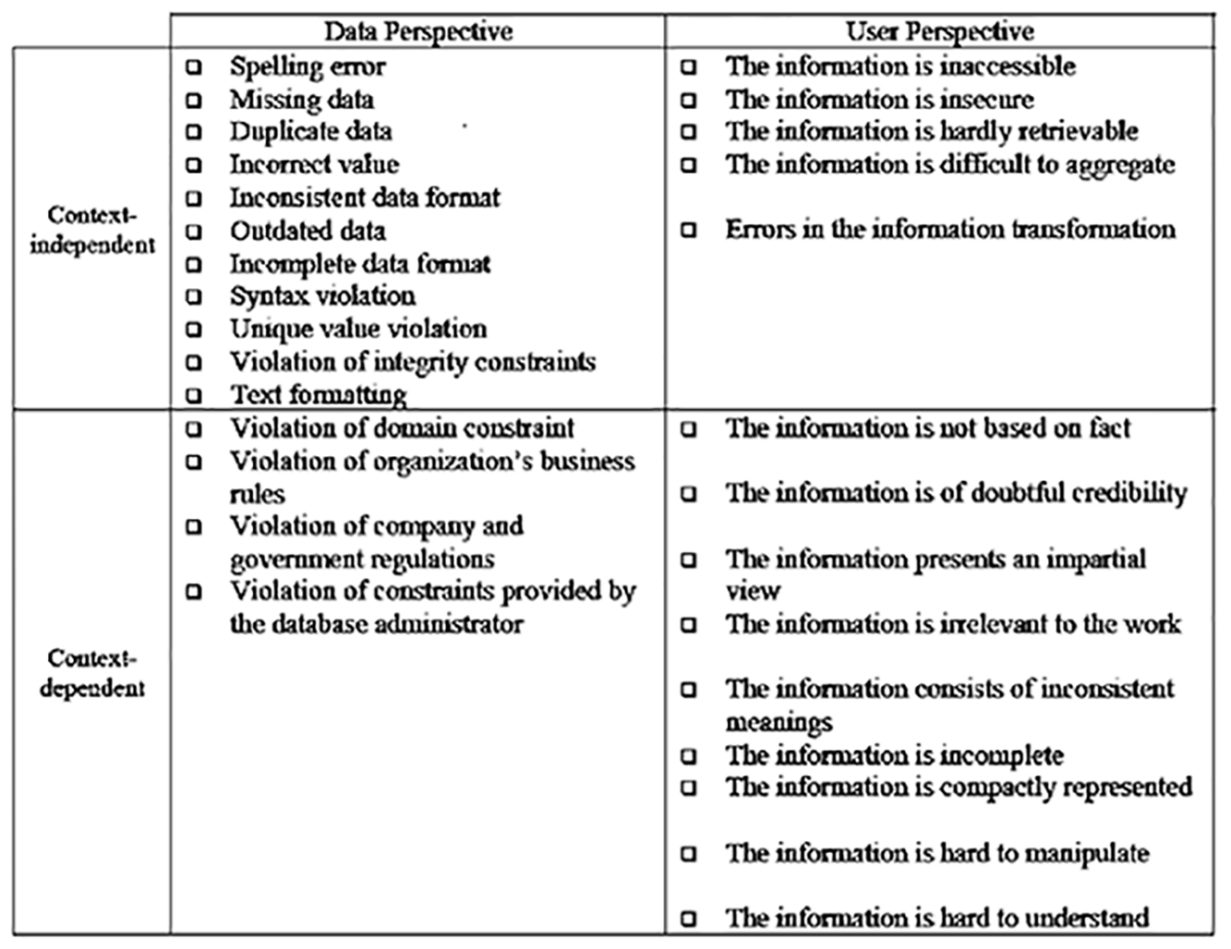
DQ Problem Classification [6].

As the framework illustrates, in order to assess DQ, a method must identify a series of dimensions and assign numerical or categorical values to them. Each dimension is a single aspect of DQ such as *accuracy*, *completeness*, *consistency*, and *timelines*. Metrics represent measurable DQ problems (DQP’s). These DQP’s are classified by a two-by-two conceptual model. The columns represent DQP’s from *data* and *user* perspectives, and the rows represent *contextual dependence* or *independence*. Fig 3 illustrated the model:

DQ problems are generally associated with one or more dimensions. For instance, data carelessly typed into a database is associated with *accuracy*. The dimensions are measured either objectively or subjectively (Pippino, Lee, & Wang, 2002). Subjective measurements are based on the extent to which data is fit for use by the consumer. For example, in [7] special questionnaires are used to measure dimensions. The questionnaire asks the value of the dimension (0–10) from four views of *definition*, *synonym*, *direct*, and *reverse*.

Objective measurements are based on the extent to which data conforms to specifications. For example, in [8]*simple ratio* is proposed which is the undesirable outcomes divided by total outcomes subtracted from 1. Eq 1 shows accuracy measurement by simple ratio in a typical database:

$$Accuracy=1-\frac{Inaccuracte.Tuples}{Total.Tuples}$$ (1)

As mentioned earlier a DQ method is a set of guidelines and techniques to define a process for DQ assessment and improvement. As the literature review section shows, many of the current methods have one or more of the following shortcomings:

1. Sometimes the instructions are too high level and do not present operational details.
2. Some methods require the help of experienced IT specialists from outside which may be against general organizational policies.
3. The cost and the volume of work for some methods are so high that most organizations may forego the potential DQ improvement benefits.
4. Some methods are designed for a specific type of organization or data system, and they lack generality.
5. DQ methods operate largely *reactively* and attempt to improve DQ after DQP’s occur and are detected.
6. Most DQ methods largely concentrate on *data-driven* strategies, meaning that they directly manipulate data in order to raise DQ. However, if the processes that access and update data are not considered, it will be only a matter of time before DQ level diminishes again.
7. Most methods do not pay close attention to the negative roles that the human agents play in creating DQP’s. Such problems are more prominent in organizations where work flows are not fully automated and are largely driven by the human agents.

In this paper we present a task based DQ method (TBDQ) which uses subjective and/or objective measurements to assess DQ. The method then attempts to improve DQ by *analyzing and modifying organizational processes* (sequence of tasks) which potentially create DQ problems. In other words, TBDQ is largely a *process-driven* method. Re-engineering the current processes in an organization could prove very expensive and also meet resistance from the organization. Hence, modifying the processes in TBDQ does not mean *altering* the current tasks, but to *add* new improving tasks to counter the effects of risky tasks. The TBDQ method is most appropriate for small and medium organization in which a sophisticated IT infrastructure is not present and human agents play an important role.

The rest of paper is organized as the following: section two reviews some of the existing DQ methods in order to show the above research gaps, section three describes the TBDQ method, section four case studies the method, finally section five concludes the paper.

## 2. Literature Review

Some of the prominent DQ methods/methodologies in the literature are the following:

*Total Data Quality Management (TDQM)*[9]draws an analogy between information quality and product quality. The method defines a new cycle based on Deming[10]which has four steps: 1.*Define*, 2.*Measure*, 3.*Analyze*, and 4.*Improve*. TDQM views information as a product, hence it claims that the principles that govern the manufacturing of products could be applied to the manufacturing of *information products*. The TDQM cycle is very practical for *continuous* improvement of DQ; however, the operational instructions are very high level and offer few details.

*A methodology for information quality assessment (AIMQ)*[11]has three main components. The first is the *product service performance model for information quality* (PSP/IQ) which is a 2 × 2 matrix[12]. The rows of the model view data either as a *product* or a *service*, and the columns view the quality of data as either *conforming to specifications* or *meeting consumer expectations*. In each quadrant of the model the associated *dimensions* are included. The second component of the AIMQ is a questionnaire-based method to measure dimensions. In a two-step survey a special questionnaire is designed and given to respondents to assess the value of the PSP/IQ dimensions. The third component performs *benchmarking* and *role gap analysis*. Benchmarking compares the organizations with best practice organizations in terms of DQ. Role gap analysis evaluates the possible discrepancies between opinions of *data users* and *information system professionals* about DQ. AIMQ is very simple and effective for DQ assessment. However, it is only a subjective assessment method and does include any improvement guidelines.

*Hybrid Information Quality Management (HIQM)* [13]attempts to manage *detection* and *correction* of errors via two cycles. The detection cycle first performs a *DQ Environmental Analysis* and then repeatedly performs *Resource Management*, *Quality Requirements Definition*, and *Strategy Correction*. The correction cycle is nested and is performed after *Quality Requirement Definition*. It performs *Quality Measurement*, *Improvement*, and *Analysis & Monitoring* which includes a warning management. HIQM is a complete method that includes both assessment and improvement. Also it benefits from a warning management module for runtime detection and improvement of DQ problems. However, the phases are not described in sufficient detail and there are no specific guidelines for implementing the phases.

In [14]a DQ framework is proposed that allows the users determine the causes of data quality problems at different levels of granularity on databases. It enables users to enhance DQ level thru monitoring and cleansing. The framework has seven steps which are performed in a cycle. The steps are: *1*. *Identification of DQ Problem*, 2.*Identificiation of Relevant Data*, *3*.*Identification of Relevant Business Rules*, *4*.*DQ Assessment*, 5.*Business Impact Determination 6*.*Cleansing of Data*, *7*.*Monitoring and Assessment on Regular Basis*. The framework is very simple to use and its quality assessment considers *data provenance*, *data fusion* and *conflict resolution* for inconsistent data. However, it is designed only for DQ projects in heterogeneous multi-database environments only.

*Heterogeneous Data Quality Methodology (HDQM)* [15]considers different types of data (structured, semi-structured, and unstructured) and takes into account only two dimensions, *accuracy* and *currency*. The method has three phases of *State Reconstruction*, *Assessment*, and *Improvement*. In the improvement phase a *Resource/Improvement-Activity* matrix is formed where the rows are general improvement activities and the columns are data resources. Each cell of the matrix is a specific case of the improvement activity for the respective data resource. To devise an improvement process a “path” thru cells of the matrix is drawn which must cover all the data resources (i.e. columns). If several paths can be drawn the one with the best improvement to cost ratio is chosen. The method is very practical in terms of providing several improvement options to choose from. However, it only considers two dimensions and there is little operational detail about forming the Resource/Improvement-Activity matrix.

*Hybrid Approach to DQ (HADQ)* [16] considers the fact that different organizations may have different requirements in the context of DQ assessment. HADQ identifies all the main assessment “activities” in some of the well-known methods, lists them, defines “dependencies” among them, and classifies each activity as *recommended* or *optional*. An organization can select from activities in order to design a *customized* assessment technique. HADQ is probably not a method by itself but offers building blocks to design one, and it does not offer DQ improvement guidelines either.

There are also other methods such as Cost-Effect of Low Data Quality (COLDQ) that view DQ from a cost-benefit point of view [17]. Other methods are designed for very specific purposes such as Canadian Institute for Health Information (CIHI) for healthcare DQ [18], Italian National Bureau of Census (ISTAT) for census [19], Information Quality Measurement (IQM) for web data [20], Quality Assessment of Financial Data (QAFD) for finance [21], Data Warehouse Quality (DWQ) for data warehousing [22], and Data Quality In Cooperative Information Systems (DaQuinCIS) for cooperative information systems [23] etc. These methods are very effective for very specific purposes, but lack generality.

There has also been research for DQ *frameworks*, *models*, and *tools*. *Information Product Map* (IP-MAP) [24]is a modeling technique that illustrates the manufacturing of information products. In [25] a *control matrix* is constructed and used to show in an organization which DQ problems have a control (or check) associated with them and how effective the controls are. The rows of the control matrix are checks and columns are DQ problems. In [26] *control charts* (or Shewhart charts) are used to monitor DQ. In [27]artificial neural networks (ANN) are used to model DQ processes that are not well-known in details. However, large number of examples is needed to train the ANN. Finally, in [28]a hierarchical framework is presented for “*big data*” and based on this framework a dynamic assessment process is constructed for DQ.

The above literature review supports the shortcomings and gaps mentioned in the previous section. In order to fill the research gaps new DQ methods must be designed which analyze and improve not the data itself, but the ***processes*** that manipulate data. There have been previous works that address DQ from a process point of view. For example, in [29]a business process modeling framework is proposed for the analysis of DQ issues. The model is extended with mathematical formulas to calculate error propagation. In [30] Business Process Modeling Notation (BPMN) [31]has been extended with new items to include DQ issues. However, such works only *model* DQ issues within processes, and do not present a method for improvement. Hence, there seems to be a need to take the first step towards a pragmatic and comprehensive method which has a *process-driven* view towards DQ and tackles DQP’s proactively at the source.

## 3. The TBDQ Method

TBDQ is mainly a *process-driven* DQ method which specially assists organizations in which people play a significant role in the creation and manipulation of data directly or indirectly. At this point TBDQ mainly focuses on "structured data", even though the significance of semi-structured and unstructured data is acknowledged by the authors. In TBDQ’s approach organizational processes are called *process units (PU’s)*. A PU is considered as a sequence of *tasks* that create, read, and update *data units (DU’s)*. A task is an activity that probably cannot be logically broken down further into smaller activities. Each PU has a specific owner who is responsible for the management and proper execution of it. A DU is a separable collection of data with well-defined contents. Each DU has an owner who is responsible for the management of DU and its DQ maintenance. Finally, a team of *Experts* is identified in the organization which is responsible for the proper planning and implementation of DQ method.

**Fig 4:**
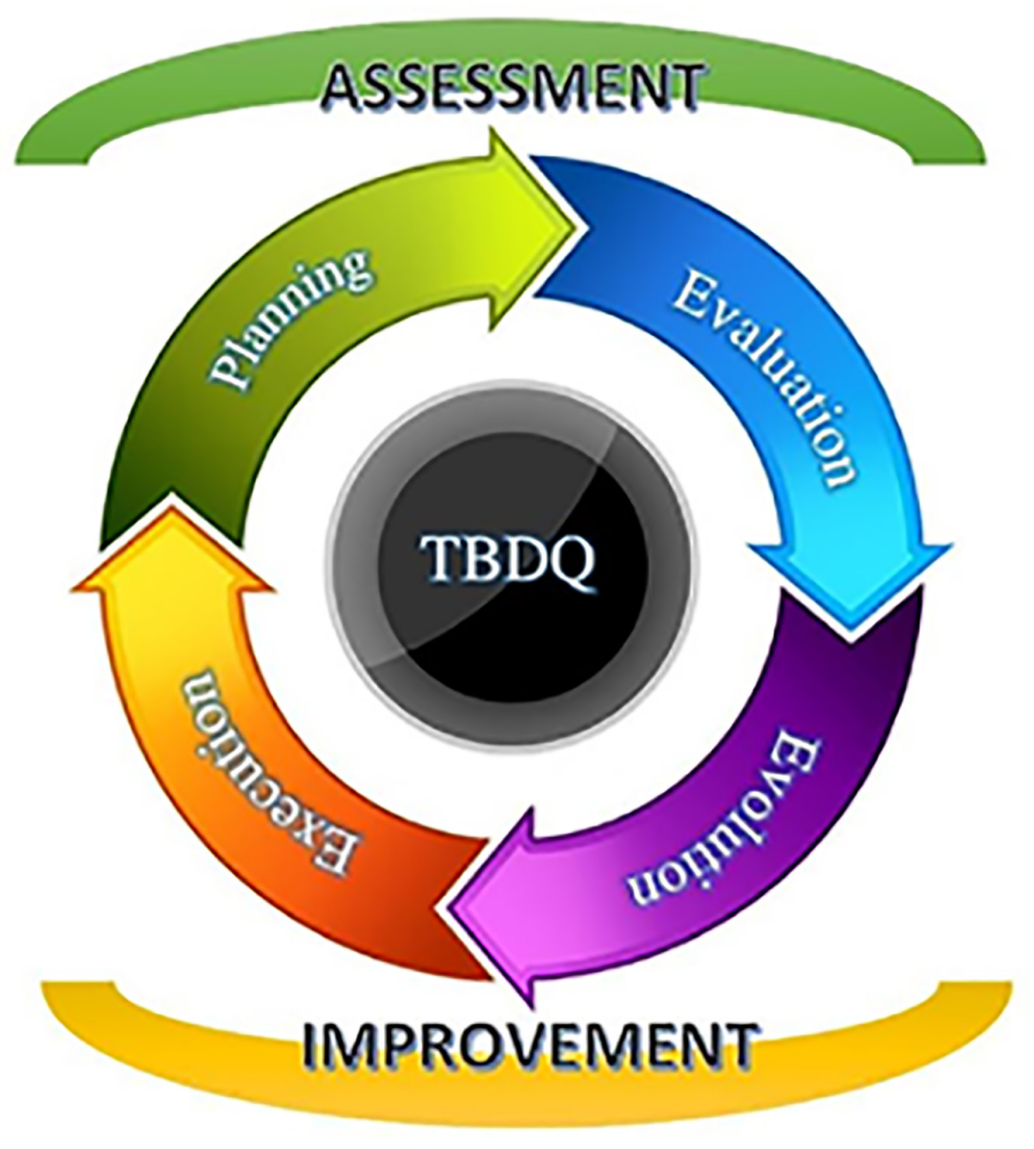
TBDQ Phases and Steps.

**Fig 5:**
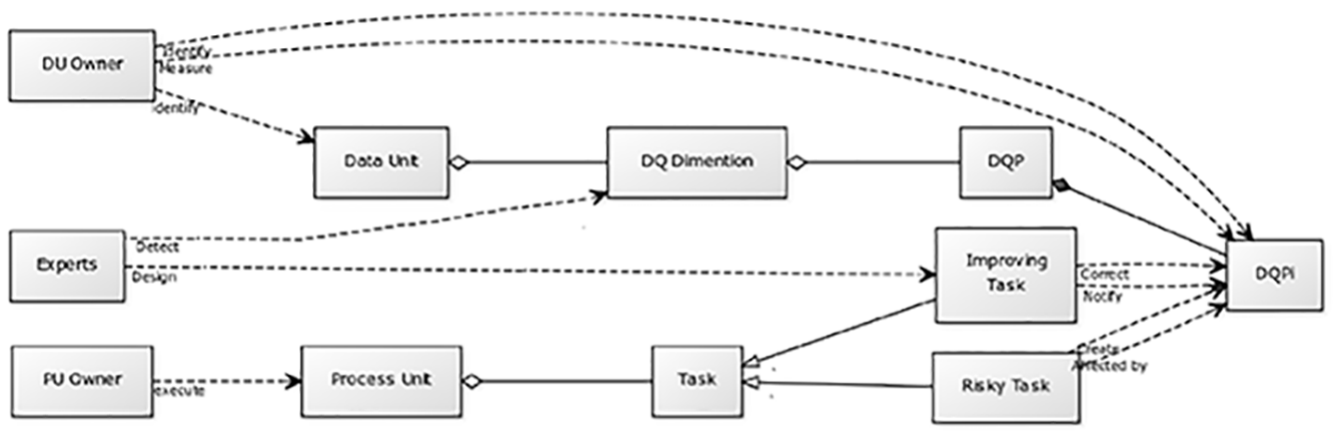
TBDQ Schema.

TBDQ has two main phases: A*ssessment* and *Improvement*. The two main phases can be repeated cyclically until the desired DQ levels are reached. The assessment phase has the following steps: *Planning* and *Evaluation*. The improvement phase has the following steps: *Evolution* and *Execution*. The activities of each phase are described in more detail in the following sections. Fig 4 shows the phases and steps:

From an operational point of view, TBDQ investigates that in each DU which specific *instances* of DQP’s (DQPi’s) exist. Hence, the method creates a mapping from DU’s to DQPi’s. Then the mapping is extended by investigating that the DQPi’s are potentially *created by* or *affect* which tasks. These are called the *risky tasks* and the associated processes are called *risky processes*. Next, new *improving tasks* are designed and inserted into the risky processes in order to counter the effects of the risky tasks hopefully improving DQ in the associated DU’s. A schematic view of TBDQ is illustrated in Fig 5:

### 3.1. The TBDQ Assessment Phase

#### 3.1.1. Planning

In this step the planning of DQ is performed. Planning involves identifying or refining the objectives and scope of DQ as well as DQ dimensions, tools, risks, scheduling, and budget. The objective of TBDQ is to raise the quality of DU’s to a specified minimum level (e.g. 80% quality). The team of Experts and DU owners can consult each other to determine the following:

1. A minimum level of DQ for each DU in the organization
2. Dimensions (DQPi’s) that matter the most for each DU (see *Identify Dimensions* activity)
3. The “weight” of each dimension (DQPi) for the respective DU (see *Evaluation* step)
4. A method of measurement for each dimension (DQPi) (see *Evaluation* step)

After measuring all the relevant dimensions in a DU, a “weighted average” of dimension values is calculated to determine the overall DQ for each DU (see Eq 2).

**Fig 6:**
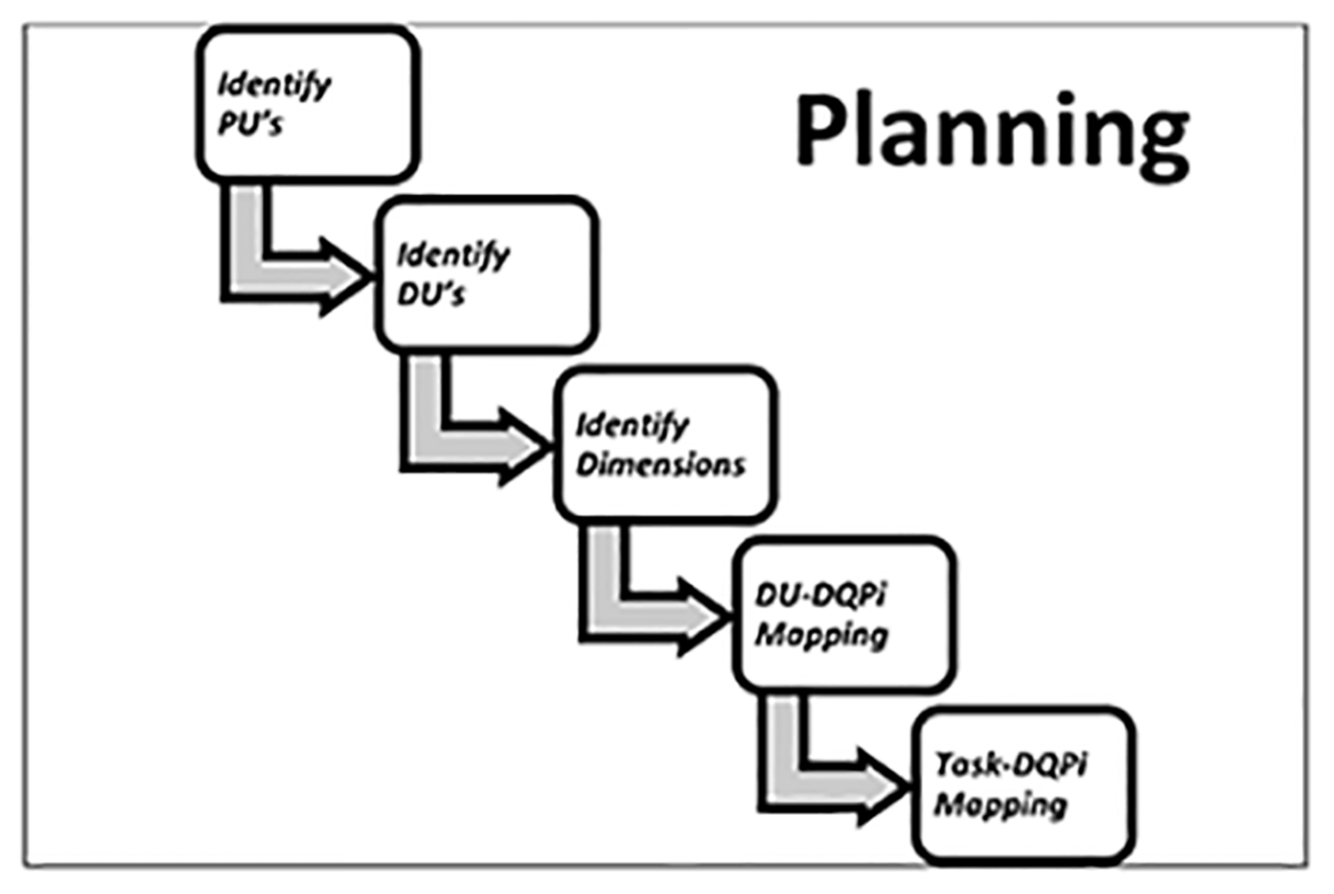
Activities of Planning.

The activities of the Planning step are illustrated in Fig 6:

*Identify Process Units*: Any typical organization has a set of specific processes which occur within known departments and units. These departments and units are probably in close interaction with each other. Identifying PU’s is an incremental task which can be done one department or unit at a time. When a department or unit is selected two sources exist that help us identify PU’s: 1) The *documents* and 2) the *workers*. Two main types of documents are considered which are:

1. *Organizational Documents* which specify general policies, procedures, organizational architecture, regulations, etc.
2. *Technical System Documents* which specify technical details of the organizational systems and processes such as use-case diagrams, sequence diagrams, activity diagrams, etc.

By studying the above documents and interviewing the workers the sequence of tasks within each PU can be identified. Also if the PU’s affect each other negatively the PU owners can send “*notifications*” to make each other aware of such situations. For instance, if PU1 writes bad data that PU2 reads, a notification is sent from PU2 to PU1.

*Identify Data Units*: Scope of DQ is the set of DU’s with quality levels less than requirements. DU’s in an organization can be identified by the PU’s and where they perform their operations such as *read*, *update*, etc. A structured DU mainly has the granularity of a database table or a spreadsheet. Identification of DU’s could be done in a top-down fashion. For example, it could start with departments, databases within a department, and finally tables within the database. To identify the DU’s a CRUD matrix is used. In this matrix the rows are the PU’s that were identified previously. The columns on the other hand are the DU’s that are *created*, *updated*, *read*, or *deleted* by the PU’s. Each cell shows the relationship between the corresponding PU and DU in the matrix. A sample CRUD matrix is shown in Table 1.

**Table 1:** CRUD Matrix.

	DU1	DU2	DU3
**PU1**	CRU	CRU	CU
**PU2**	RD	URD	RD
**…**	…	…	…

*Identify DQ Dimensions*: The most widely used dimensions in each organization are *accuracy*, *completeness*, *consistency*, and *timeliness* [32]. However, there could be more dimenions that specifically matter to an organization [1]. A questionnaire based method could be used to identify the most relevant dimensions [33] for each DU in the organization. For each dimension associated DQP’s and DQPi’s are determined to make method more specific for the organization at hand as it will be seen in the next activity.

*Design the DU-DQPi Mapping Table*: Each row of the table identifies which DU has which problematic dimension, as well as the associated DQP and DQPi. The data users of a DU are asked whether a dimension is problematic in their DU (e.g. accuracy). If the answer is ‘yes’ one or more associated DQP’s must be identified for the problematic dimension. If the answer is ‘No’ the dimension is not problematic for the DU, and nothing needs to be done. The DQP’s are selected from the first quadrant of Fig 3. The first quadrant is used because TBDQ views DQ problems from the *perspective of data units* and is designed to be *context-independent*. Also to make the method more specific for the organization for each DQP one or more *instances* (DQPi’s) are identified. DQPi’s are very specific cases of DQP’s. A sample DU-DQPi mapping is shown in Table 2.

**Table 2:** DU-DQPi Mapping.

DU	Dimension	DQP	DQPi
DU1	Accuracy	Spelling Error	Some last names in the database are misspelled
DU2	Completeness	Missing Data	Some email addresses are missing from the database
DU2	Consistency	Duplicate Data	Some customer names are repeated more than once in the database
DU3	Timeliness	Outdated data	Some phone numbers are no longer valid in the database.
…	…	…	…

*Design the Task-DQPi Mapping Table*: In this step it must be specified which task from each PU might create a DQPi. To achieve this CRUD matrix and the DU-DQPi mapping table must be used together. The main idea behind this activity is that DQPi’s are generated when data is *created*, *updated* or *read*. If data is erroneously created or updated, eventually another task reads the erroneous data and will be negatively affected by it. Hence, *create* and *update* tasks generate DQPi’s, while *read* tasks are affected by DQPi’s. Experience shows that the majority of DQPi’s are generated at the time of data creation, where large data entry operations are prone to a certain percentage of errors. Notice that a *deleting task* cannot possibly generate a DQPi because associated data is erased and will never be read. Hence, delete operations are not used in the CRUD matrix in TBDQ. The mapping table is designed as the following:

For each “*C”* or *“U”* from the CRUD, it is evaluated if the related task potentially *generates* a DQPi in the DU. If the answer is ‘yes’ the associated task is considered risky (the related PU is considered a Risky PU) and the DQPi is associated with the creating or updating tasks.

Also for each *“R”* from the CRUD, it is evaluated whether the task is *affected by* DQPi’s in the DU. If the answer is ‘yes’ the associated task is considered risky (the related PU is considered a Risky PU) and the DQPi is associated with the read. Readings might be affected by a creating or updating that has been performed *inside or outside* of the PU. Creating and updating tasks inside and outside of PU probably require different improving tasks.

At the end a matrix is formed in which each row identifies which PU’s risky tasks create or are affected by which DQPi’s. A sample is shown in Table 3.

**Table 3:** Task-DQPi Mapping.

*Task*	*Is Risky*	*Update/Read*	*DQPi*
***PU1*.*Task1***	*yes*	*C*	*DQPi 2*
***PU1*.*Task2***	*no*		
***PU1*.*Task2***	*yes*	*R*	*DQPi 3*
**…**			…
***PU2*.*Task1***	*yes*	*R*	*DQPi 2*
***PU2*.*Task1***	*yes*	*U*	*DQPi 1*
***PU2*.*Task1***	*no*		
…	…	…	…

**Fig 7:**
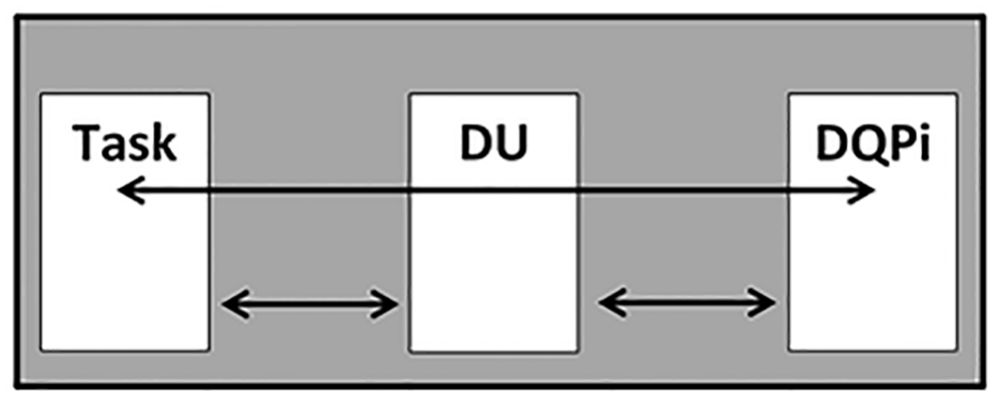
Three Mappings in TBDQ.

Now consider Tables 1, 2 and 3 together. There is a mapping from tasks to DU’s, a mapping from DU’s to DQPi’s, and a mapping from tasks to DQPi’s. Please see Fig 7:

The red arrow is significant because it avoids the problem of *loop traps*. Now a combined mapping may be created where it is determined which task is associated to which DQPi in which DU. Table 4 illustrates such a mapping.

**Table 4:** Task-DQPi Table.

*Task*	*Is Risky*	*Update/Read*	*DU*	*DQPi*
***PU1*.*Task1***	*Yes*	*C*	*DU2*	*DQPi 2*
***PU1*.*Task2***	*No*		*DU1*	
***PU1*.*Task2***	*Yes*	*R*	*DU2*	*DQPi 3*
**…**	…	…	…	…
***PU2*.*Task1***	*Yes*	*R*	*DU1*	*DQPi 2*
***PU2*.*Task1***	*Yes*	*U*	*DU2*	*DQPi 1*
***PU2*.*Task1***	*No*		*DU3*	
**…**	…	…	…	…

Table 4 is later used in the method to design improving tasks.

#### 3.1.2. Evaluation

**Fig 8:**
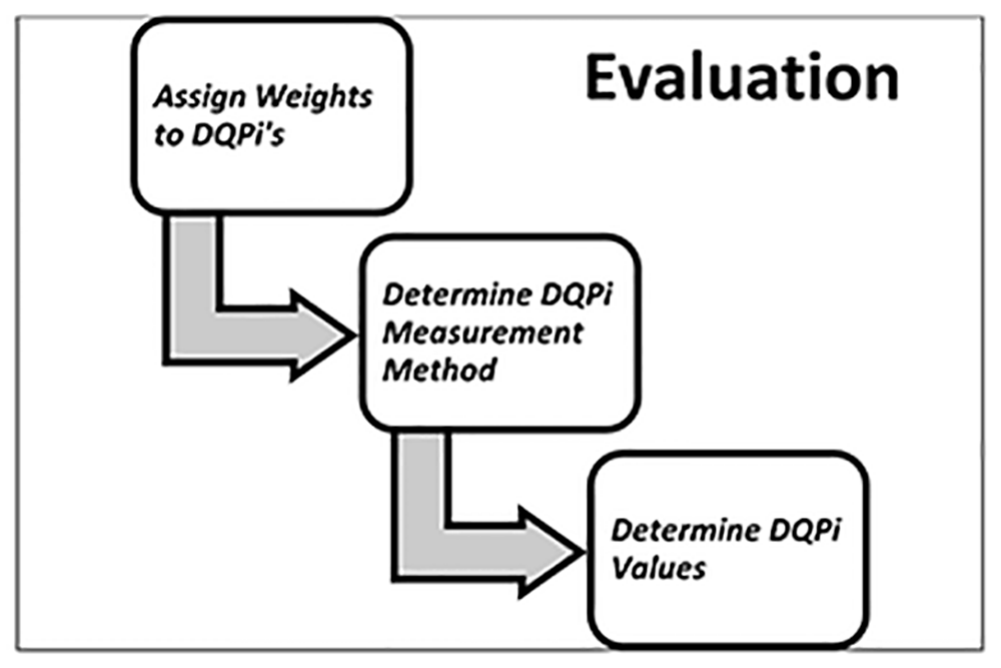
Activities of Evaluation.

The activities of the Evaluation step are illustrated in Fig 8:

*Assign Weights to DQPi’s in each DU*: Each DQPi in a DU has a certain *weight* with respect to the other DQPi’s in the same DU. The Experts team and DU owners can consult each other to determine the weight of each DQPi using pair-wise comparison matrix of Analytical Hierarchical Process (AHP) [34].

*Determine DQPi measurement method*: For each DQPi it is determined which measurement method must be used. For subjective measurement TBDQ uses a questionnaire based method [7]and for objective measurements the method uses *simple ratio* [8]. For each DQPi, objective, subjective or both measurements may be used, depending on the nature of DU and DQPi.

*Determine DQPi Values*: In this step the team of Experts and DU owners can cooperate to measure the DQPi’s using measurement methods determined in the previous activity. For example, by simple ratio if a DQPi is observed twenty percent of the time by DU users, its measurement is 0.8 (i.e. (1–0.2) / 1). The DQPi measurements are expected to be reliable. One way to determine that is to see if subjective and objective measurements are in reasonable agreement with each other.

### 3.2. The TBDQ Improvement Phase

#### 3.2.1. Evolution

**Fig 9:**
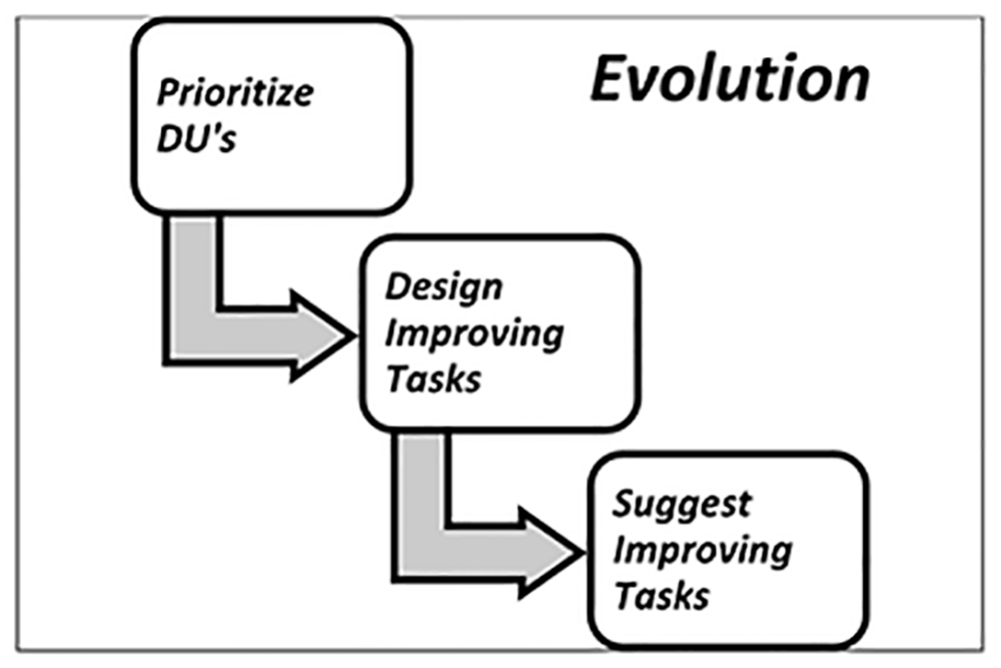
Activities of Evolution.

The activities of the Evolution step are illustrated in Fig 9:

*Prioritize DU’s*: The DU’s are prioritized according to *DU Quality* (*DuQ)* values. The DuQ is calculated based on the measurement and weight of all the DQPi’s within the related DU. Eq 2 shows the calculation:

$$DuQ(DU_{j})=\sum_{DQP_{i}\in DU_{j}}Weight(DQP_{i})\times Measurement(DQP_{i})$$ (2)

tice that the lower the value of DuQ the higher the priority. Of course, there are other parameters that could affect the priority of a DU such as the opinion of the Experts team and DU owners, organizational business strategies, DU significance, etc. Eventually, a list of DU priorities is achieved at the end of this step.

In addition to prioritization, the DuQ values can be compared to values from the previous cycle of the TBDQ to see how the overall improvement of the DU’s has been.

*Design Improving Tasks*: In order to counter the effects of risky tasks, a set of new improvement tasks are designed for each DQPi and inserted into the respective PU. The team of Experts, DU Owners, and PU owners consult each other to design the improving tasks. In TBDQ improving tasks are mainly *data correction* or *notification*. For instance, if the DQPi is an instance of *Duplicate Data*, a data correction task would to remove duplicate data, and notification task would be to send a notification to the updating agent in order to prevent the duplicate data.

The Task-DQPi table helps identify which *risky task* creates which DQPi, hence, designing an *improving task* is clearer. If the risky task is an *update* some data correction must designed for the risky update. Since the data correction must be performed before the updated data is *read*, the improving task is inserted into the PU immediately after the update or as close to it as possible. If the risky task is a *read*, there are several different situations. If the associated update is within the current PU a data correction is done. If the associated update is from another PU, a notification is sent to the PU’s owner, and the respective PU is added to the scope of PU’s being evaluated in the next TBDQ cycle. If the update is from outside of the organization only a notification is sent to the external party. Notice that covering all human errors in DQ is not possible at this juncture, however, by utilizing controls and safety procedures in the form of improving tasks, the risky tasks may be controlled considerably.

**Fig 10:**
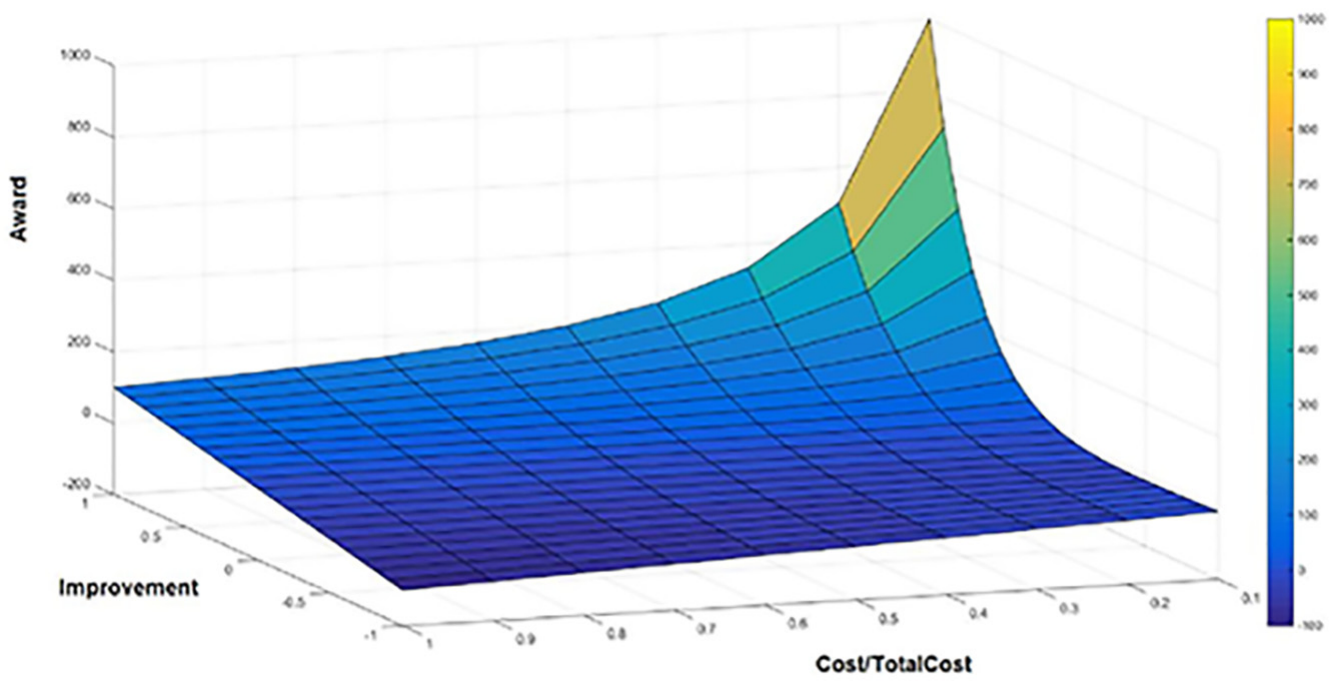
Award representation by relative cost and DQPi improvement.

*Suggest appropriate Improving Tasks*: The designed improving tasks can now be implemented in the risky PU’s. The Expert team can suggest which improving tasks be implemented if there is more than one for a DQPi. Also several could be tested together so see a combined effect on a DQPi. Due to insufficient knowledge, in the first implementations the suggestion of improving tasks is only based on the opinion of the Experts or trial-and-error. However, TBDQ has an “*award system*” that helps Experts to suggest improving tasks more logically in the next TBDQ cycles. The award system analyzes improving tasks and assigns an *award* value based on two main components which are 1) *execution cost* and 2) *the level of improvement on the DQPi*. The cost of a task is measured relative to the total cost of the PU to which it belongs. In order to measure the cost of individual tasks *Time-Driven Activity-Based Costing* (TDABC) is used[35]. Eqs 3 and 4 define TBDQ’s award and improvement functions respectively. Also Fig 10 illustrates how the award value changes as a function of cost and improvement.

$$Award(Task_{i},DQP_{k})=100\times Improvement(DQP_{k})\times {\left(\frac{Cost(Task_{i})}{\underset{for.all.Task_{j}.in.Taski.PU's.sequence}{\sum }Cost(Task_{j})}\right)}^{-Improvement(DQPk)}$$ (3)

$$Improvement(DQP_{i})=-(Measure_{after}(DQP_{i})-Measure_{before}(DQP_{i}))$$ (4)

In Eq 3 the relative cost is raised to the power of minus improvement, because a higher award must be given to a less costly task in both cases of positive or negative improvement. For example, consider Task1 and Task2 with relative costs of 1/10 and 9/10 respectively. If the improvement is 0.5, the former gives an award of 158.1 and the latter an award of 52.7 which means Task1 is more beneficial than Task2. However, if the improvement is -0.5 the awards for Task1 and Task2 are -15.8 and -47.4 respectively, which means Task1 generates less loss than Task2.

Also if more than one improving task is assigned to a single DQPi, it is unknown which task contributed how much to the improvement. Hence, the assumption is made that all tasks contributed equally. For instance, if Task_i_ and Task_j_ together caused an improvement of 0.5 for DQPi_k_ the contribution for each task is assumed to be 0.25. To better evaluate the individual contributions the improving tasks must be executed individually.

Fig 10 shows the award value as a function of cost and improvement.

#### 3.2.2. Execution

**Fig 11:**
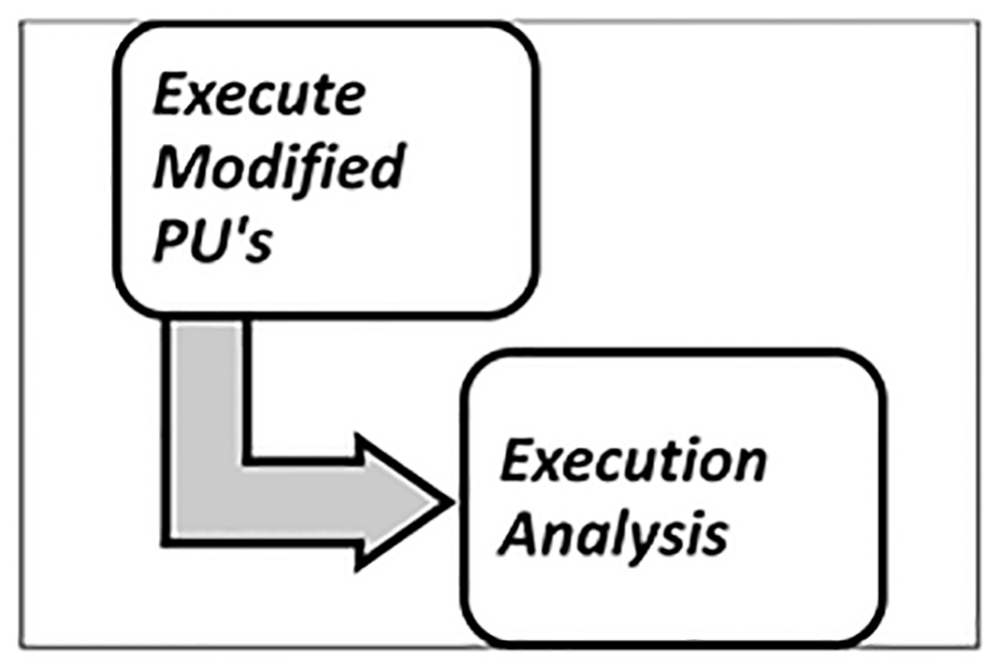
Activities of Execution.

The activities of the Execution step are illustrated in Fig 11:

*Execute the modified PU’s*: In this activity PU’s with the suggested improving tasks are executed. The Experts team and the PU owners determine where in PU’s the new tasks are inserted. Also enough time must be given to the improving task to show their potential effects. The extent of this time is also determined by Experts and the PU owners. Depending on the organizational circumstances this time period could vary considerably.

*Analysis of the Execution*: As mentioned earlier the improving tasks are either notification or data correction. In case a notification is sent to another PU it must be checked to see if that PU is within the current scope of data quality. If it is not, in the next planning step this PU must be added to the scope. Of course, this can only be done if the notified PU is within the organization. Otherwise, sending a notification is all that can be done. In case of data correction if any improvement regarding the DQPi’s is achieved, the amount of improvement will be assessed in the next evaluation step, and incorporated into the next award calculation.

### 3.3. A Model for Analysis of Data Quality

**Fig 12:**
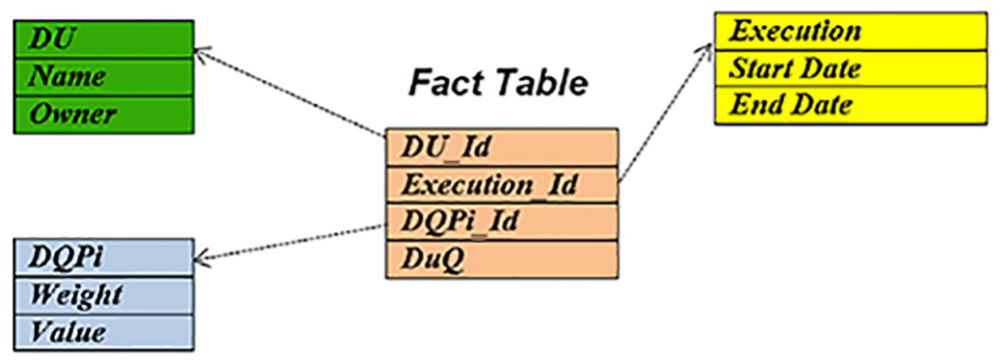
Star Schema for TBDQ.

To better analyze the overall effect of TBDQ on DQ in an organization a model is needed. Here we benefit from *star schema*. The ultimate goal of TBDQ is to raise the DQ in each *DU* by improving the *DQPi’s* after each *execution*. Hence, three dimensions are considered which are DU, DQPi, and Execution. The fact table consists of the ID’s for these three dimensions as well as DuQ measurement (see Eq 2) after the corresponding execution. The DuQ values are used for overall DQ analysis. The star schema architecture is illustrated in Fig 12.

In effect what the above model dictates is to measure DuQ values in order to have an overall assessment of how DQ is changing in the organization. With this model, it could be analysis the measure of the Data Quality in each Du, in each DQPi, or in each Execution.

## 4. Case Study

TBDQ’s effectiveness and applicability was tested through a case study. The case study was done in Behta, an international seed trade company. The complete board of directors including the directing manager of the company signed a written consent approving the case study in the company. The agreement was that the company would cooperate with the study and in return it will use the potential benefits and results of the study. All participants signed an informed written consent expressing that they were willingly participating in the case study. The data was made available to the researchers through company employees. All the data were accessed legally and with complete consent of all the relevant parties at Behta. No personal information was taken from any of the Behta employees or customers. The employees were asked not to include names, family names or any other personal information in the questionnaires, database tables, or any other data that were used in the research. Also the authors took care to delete any such information if they were revealed to them by accident.

### 4.1. Behta Introduction

The Behta Company has been importing and distributing seeds in Iran for the last thirty years. In order to distribute seeds first it has to be imported in “sample amounts” and tested for the local soil and climate. The testing of sample seeds in the farms could take as long three years. After successful testing, the seeds are imported in commercial amounts and distributed among the farmers. The company currently represents several foreign suppliers, and has a pool of about 3000 nation-wide representatives and customers. The company was selected for the case study mainly because of the following reasons:

1. This is a mid-size business not too big and not too small for the case study. A very large company may prove too difficult for a novel DQ method. Also a very small company may make the case study results questionable.
2. In the company there are many employees with low or medium knowledge of IT who often use traditional business tools. Hence, DQ is prone to human error which makes for an ideal environment for TBDQ.

### 4.2. Initial Assessment Phase in Case Study

#### 4.2.1. Planning

***Identify Process Units and* the *Relevant Tasks*:**

In this step three main PU’s are identified and some of their related tasks are shown in Table 5.

**Table 5:** Company PU’s and related Tasks.

*PU*	*Technical Process*	*Sales Process*	*Customer management*
*Task*			
***1***	*Technical team orders new sample seeds for testing*	*Issue Commercial order to foreign suppliers*	*The contact information are obtained by the Sales or Technical Dept*.
***2***	*Sample Seed is received from the foreign suppliers*	*Customs agent contacts the sales dept*. *about the new shipment*	*The contact information is entered in the local copy of the Customers’ Database*
***3***	*Sample seeds information is entered in the technical database*	*Bill of lading for the new shipment arrives at the sales dept*.	*The two copies of the Customers’ Database are synchronized*.
***4***	*Technical team visits the farms to introduce new varieties to farmers and also check the progress of previous verities*	*Sales dept*. *receives the shipment at the warehouse*	
***5***	*In case*, *new farmers or customers are met during the visits their information is sent to the customers’ database*	*Sales dept*. *enters the new shipment into the commercial database*	
***6***	*Sample requests are received from farmers*	*Customers are contacted about the new shipment*	
***7***	*New Customers are Entered in the Sample Database*	*Money is deposited by the customers to the sales account*	
***8***	*Requested seed amount are deducted from the technical database*	*Invoice is issued*	
***9***	*Sample seeds are sent to the requesting farmers*	*Seed is sent to the customers and deducted from the Commercial database*	
***10***		*All customers’ accounts are also recorded manually on paper*	

*Technical Process*: involves importing and testing sample seeds in order to determine which ones are suitable for commercial purposes.

*Sales Process*: involves import and distribution of commercial seeds in large quantities.

*Customer management*: collects and maintains the information of all the customers.

***Identify Data Units:***

*Sample database*: This is the database where all the records of sample seeds are stored. These are mainly the seeds that are in the testing stage and have not been commercialized yet. Besides general seed information, the database also includes data about test results and farmers’ responses about samples.

*Commercial database*: This is the database where commercial seeds are kept. In fact, this is the main inventory of the company. The number of records is high because commercial seeds from years ago may still be in the database.

*Customers’ Database***:** This database contains the names, phone numbers, addresses, and other contact information about company customers. The database also contains business related information such how long the customer has been working with the company, his credit line, possible debt, annual sales, favorite products, etc.

Table 6 shows the CRUD matrix for Company PU’s and DU’s. The PU’s perform the four CRUD operations for their respective database. Also sales and technical processes read from the customers’ database to obtain their contact and credit related information. Sometimes while visiting farms the technical team finds new customer farmers whose information must be entered into the customers’ database.

**Table 6:** Company CRUD Matrix.

	*Sample Database*	*Commercial Database*	*Customers’ Database*
***Technical Process***	*CRUD*		*UR*
***Sales Process***		*CRUD*	*R*
***Customer management***			*CRUD*

***Identify DQ Dimensions:***

A questionnaire based survey was done in the company to determine the most significant dimensions [33]. The survey revealed that the following dimensions carried the most significance for data users: *accuracy*, *completeness*, *consistency*, and *timeliness*. This is consistent with results from [32]. In our survey questionnaire one "item" was included for each dimension, in the form of the dimension definition. Perhaps in future trials several "items" could be included for each dimension, as proposed in [7], to see if better survey results can be achieved.

***Design the DU-DQP Mapping Table*:**

By interviewing the data users in the company it was revealed which dimensions are problematic in which DU’s. For each dimension the associated DQP and DQPi’s were also identified. The results are shown in Table 7.

**Table 7:** Company DU-DQPi Mapping Table (Separated by DU’s).

*DU*	*Dimension*	*DQP*	*DQPi*	*DQPi Description*
***Sample Database***	*Consistency*	*Syntax Violation*	*Consistency of Grams/Grain*	*The sample seeds are sometimes packed in “grams” and sometimes in number of “grains”*.
***Sample Database***	*Accuracy*	*Incorrect value*	*Accuracy of Sample Data Base*	*Sometime the database reports the presence of sample seed*, *whereas if fact none is left*.
***Sample Database***	*Consistency*	*Duplicate Data*	*Duplicate Names*	*In case of searching customers*, *if two last names are similar it is difficult to distinguish them from each other*
***Commercial Database***	*Accuracy*	*Incorrect value*	*Readable Bank Slip*	*When the bank deposit slips are faxed*, *they are very hard to read*
***Commercial Database***	*Accuracy*	*Incorrect value*	*Accuracy of Sales Paper Records*	*Since many growers are not used to computers yet*, *sales data are recorded both digitally and on paper*. *Sometimes the digital and paper recordings do not match*.
***Commercial Database***	*Accuracy*	*Incorrect value*	*Accuracy of Bill of Lading*	*Bills of lading from foreign suppliers are sometimes erroneous or incomplete*, *in terms of number of boxes*, *total number of seeds*, *etc*.
***Customers’ Database***	*Accuracy*	*Incorrect value*	*Accuracy of Customers’ Database*	*The contacts information in the real world is often changing due to change of address*, *death*, *etc*, *which may go unnoticed in the company*
***Customers’ Database***	*Completeness*	*Missing data*	*Completeness of Customers’ Database*	*For some contacts some information are missing such as email address*, *mobile phone numbers*, *etc*.

***Design the Task-DQPi Mapping Table:***

In this activity PU’s were evaluated to see which updating tasks within them create a DQPi, and which reading tasks are affected by a DQPi. In both cases the tasks are considered risky. Tables 8, 9 and 10 illustrate Task-DQPi mapping in Company for the three PU’s.

**Table 8:** Company Task-DQPi Mapping Table (Technical Process).

*PU*	*Technical Process*	*Update/Read*	*DU*	*Risky*	*DQPi*
***Task***					
***1***	*Technical team orders new sample seeds for testing*			*no*	
***2***	*Sample Seed is received from the foreign suppliers*	*R*	*Sample Database*	*yes*	*Consistency of Grams/Grain*
***3***	*Sample seeds information is entered in the technical database*	*U*	*Sample Database*	*yes*	*Accuracy of Sample Data Base-Duplicate Names*
***4***	*Technical team visits the farms to introduce new varieties to farmers and also check the progress of previous verities*			*no*	
***5***	*In case*, *new farmers or customers are met during the visits their information is sent to the customers’ database*	*U*	*Customers Database*	*yes*	*Accuracy of Customers’ Database-Completeness of Customers’ Database*
***6***	*Sample requests are received from farmers*			*no*	
***7***	*Requested seed amount are deducted from the technical database*	*U*	*Sample Database*	*yes*	*Accuracy of Sample Data Base-Duplicate Names*
***8***	*Sample seeds are sent to the requesting farmers*	*R*	*Customers Database*	*yes*	

**Table 9:** Task-DQPi Mapping Table (Commercial Process).

*PU*	*Sales Process*	*Update/Read*	*DU*	*Risky*	*DQPi*
***Task***					
***1***	*Issue Commercial order to foreign suppliers*			*no*	
***2***	*Customs agent contacts the sales dept*. *about the new shipment*			*no*	
***3***	*Bill of lading for the new shipment arrives at the sales dept*.			*no*	
***4***	*Sales dept*. *receives the shipment at the warehouse*			*no*	
***5***	*Sales dept*. *enters the new shipment into the commercial database*	*R*	*Commercia lDatabase*	*yes*	*Accuracy of Bill of Lading*
***6***	*Customers are contacted about the new shipment*	*R*	*Customers Database*	*yes*	
***7***	*Money is deposited by the customers to the sales account*	*U*	*Commercia lDatabase*	*yes*	*Readable Bank Slip*
***8***	*Invoice is issued*			*no*	
***9***	*Seed is sent to the customers and deducted from the Commercial database*			*no*	
***10***	*All customers’ accounts are also recorded manually on paper*	*U*	*Commercial Database*	*yes*	*Accuracy of Sales Paper Records*

**Table 10:** Task-DQPi Mapping Table (Customers’ Database).

*PU*	*Customer management*	*Update/Read*	*DU*	*Risky*	*DQPi*
***Task***					
***1***	*The contact information are obtained by the Sales or Technical Dept*.			*no*	
***2***	*The contact information is entered in the local copy of the Customers’ Database*	*U*	*Customers Database*	*yes*	*Accuracy of Customers’ Database—Completeness of Customers’ Database*
***3***	*The two copies of the Customers’ Database are synchronized*.			*no*	

#### 4.2.2. Evaluation

*Assign Weights to DQPi’s in each DU*: DU owners were consulted and pair-wise comparison of AHP was used to determine the weights of the DQPi’s in each DU. Tables 11, 12 and 13 show the pair-wise comparison values and weights of the DQPi’s in the three DU’s.

**Table 11:** Pair-wise Comparison Matrix for Sample Database.

	*Consistency of Grams/Grain*	*Accuracy of Sample Data Base*	*Duplicate Names*	*Calculated DQPi Weights*
***Consistency of Grams/Grain***	*1*	*1/3*	*1/2*	*0*.*33*
***Accuracy of Sample Data Base***		*1*	*2*	*1*
***Duplicate Names***			*1*	*0*.*5*

**Table 12:** Pair-wise Comparison Matrix for Commercial Database.

	*Accuracy of Bill of Lading*	*Readable Bank Slip*	*Accuracy of Sales Paper Records*	*Calculated DQPi Weights*
***Accuracy of Bill of Lading***	*1*	*1/3*	*1*	*0*.*33*
***Readable Bank Slip***		*1*	*3*	*1*
***Accuracy of Sales Paper Records***			*1*	*0*.*33*

**Table 13:** Pair-wise Comparisons Matrix for Customers’ Database.

	*Accuracy of Customers’ Database*	*Completeness of Customers’ Database*	*Calculated DQPi Weights*
***Accuracy of Customers’ Database***	*1*	*2*	*1*
***Completeness of Customers’ Database***		*1*	*0*.*5*

***Determine DQPi measurement approach:***

For all DQPi measurements in the case study *simple ratio* was used, because it was very practical for all DQPi’s. Some initial subjective measurements showed that they were rather time-consuming in the company and sometimes generated inconsistent results.

***Determine DQPi Values:***

The initial measurement of the DQPi’s are illustrated in Table 14.

**Table 14:** Initial DQPi Measurements.

*DU*	*DQP*	*Value*
*Sample Database*	*Consistency of Grams/ Grain*	*0*.*5*
*Sample Database*	*Accuracy of Sample Data Base*	*0*.*8*
*Sample Database*	*Duplicate Names*	*0*.*9*
*Commercial Database*	*Accuracy of Bill of Lading*	*0*.*8*
*Commercial Database*	*Readable Bank Slip*	*0*.*5*
*Commercial Database*	*Accuracy of Sales Paper Records*	*0*.*85*
*Customers’ Database*	*Accuracy of Customers’ Database*	*0*.*8*
*Customers’ Database*	*Completeness of Customers’ Database*	*0*.*7*

### 4.3. Initial Improvement Phase in Company Case Study

#### 4.3.1. Evolution

***Prioritize DU’s:***

The DU’s were prioritized according to Eq 2. The results are illustrated in Table 15.

**Table 15:** DU Priorities in Company.

*DU*	*Priority*
*Sample Database*	*1*.*42*
*Commercial Database*	*1*.*04*
*Customers’ Database*	*1*.*15*

As Table 15 shows the highest priority goes to the Commercial Database followed by the Customers’ Database and the Sample Database.

***Design Improving Tasks:***

The company DU owners, PU owners and Experts consulted each other to design one or more improving tasks for each risky task. Tables 16, 17 and 18 illustrate the improving tasks for the three company processes. The types of improving tasks are also included in the tables.

**Table 16:** Designing Improving Task for Technical Process.

*Task*	*Update/Read*	*DQPi*	*Improving Task*	*Improving Task Description*	*Imp*. *Task Type*
*Technical team orders new sample seeds for testing*					
*Sample Seed is received from the foreign suppliers*	*R*	*Consistency of Grams/Grain*	*remind suppliers about grams / grains*	*Remind suppliers about units by phone*, *fax*, *or email when sample seeds are ordered*.	*Notification*
*Sample seeds information is entered in the technical database*	*U*	*Accuracy of Sample Data Base*	*Corrective records*	*If a sample seed in DB does not match the inventory amount a corrective record is added to make them match*.	*Data Correction*
			*Inventory Check*	*Perform a complete inventory check to match sample DB values against actual values*.	*Date Correction*
*Technical team visits the farms to introduce new varieties to farmers and also check the progress of previous verities*					
*In case*, *new farmers or customers are met during the visits their information is sent to the customers’ database*	*U*	*Accuracy of Customers’ Database*	*Notification*: *Double-check new customers’ information*	*Customer Management PU sends a notification to technical PU to double check new customers’ information*	*Notification*
		*Completeness of Customers’ Database*			
*Sample requests are received from farmers*					
*Requested seed amount are deducted from the technical database*	*U*	*Accuracy of Sample Data Base*	*Corrective records*	*If a sample a seed in DB does not match the inventory amount a corrective record is added to make them match*.	
			*Inventory Check*	*Perform a complete inventory check to match sample DB values against actual values*.	
*Sample seeds are sent to the requesting farmers*					

**Table 17:** Designing Improving Task for Commercial Process.

*Task*	*Update/Read*	*DQPi*	*Improving Task*	*Improving Task Description*	*Imp*. *Task Type*
***Issue Commercial order to foreign suppliers***					
***Customs agent contacts the sales dept*. *about the new shipment***					
***Bill of lading for the new shipment arrives at the sales dept*.**					
***Sales dept*. *receives the shipment at the warehouse***					
***Sales dept*. *enters the new shipment into the commercial database***	*R*	*Accuracy of Bill of Lading*	*remind suppliers about bill of lading*	*Remind suppliers about bill of lading by phone*, *fax*, *or email*, *when commercial seeds are ordered*.	*Notification*
***Customers are contacted about the new shipment***					
***Money is deposited by the customers to the sales account***	*U*	*Readable Bank Slip*	*Hire a bank mediator*	*Hire a person to check all deposit at the bank*.	*Data Correction*
			*Internet banking*	*Use internet banking to confirm all deposits at the bank*.	*Data Correction*
***Invoice is issued***					
***Seed is sent to the customers and deducted from the Commercial database***					
***All customers’ accounts are also recorded manually on paper***	*U*	*Accuracy of Sales Paper Records*	*Hire a sales agent*	*Hire a sales agent to re-confirm paper recordings*	*Data Correction*

**Table 18:** Designing Improving Task for Customer Management.

*Task*	*Update/ Read*	*DQPi*	*Improving Task*	*Improving Task Description*	*Imp*. *Task Type*
***The contact information are obtained by the Sales or Technical Dept*.**					
***The contact information is entered in the local copy of the Customers’ Database***	*U*	*Accuracy of Customers’ Database*	*Visiting technical team checks the customer information*	*While visiting farms and representatives the technical team is required to re-check the contact information*.	*Data Correction*
		*Completeness of Customers’ Database*	*Instant correction of out of date information*	*If contact information proved outdated or incorrect*, *the contacting party is required to do the research and correct it*.	*Data Correction*
***The two copies of the Customers’ Database are synchronized*.**					

Notice that the technical and commercial processes “*read*” from the customers’ database. However, the reading tasks are not included in the above tables because they are performed very frequently and also the associated DQPi’s (i.e. accuracy and completeness of the customers’ database) is properly dealt with in the customer management process.

***Suggest Improving Tasks:***

For each risky task one or more improving tasks were selected by the Experts teams and inserted into the PU’s after consulting PU owners. In order to obtain awards the costs of the tasks were also calculated. The costs of tasks were measured $/month, based employee time and other company resources spent on the task monthly, such as internet, telephone, package delivery, etc. The new PU’s along with the tasks’ costs are illustrated in Tables 19, 20 and 21.

**Table 19:** Improving Tasks and Costs for Technical Process.

*PU*	*Technical Process*	*Cost*
***Task***		
***1***	***Remind suppliers about grams / grains***	***50***
*2*	*Technical team orders new sample seeds for testing*	*200*
*3*	*Sample Seed is received from the foreign suppliers*	*100*
*4*	*Sample seeds information is entered in the technical database*	*150*
*5*	*Technical team recommends new varieties to farmers*	*400*
*6*	*Requests are received from farmers*	*300*
*7*	*Requested seed amount are deducted from the technical database*	*150*
*8*	*Sample seeds are sent to the requesting farmers*	*700*
***9***	***Corrective records***	***150***

**Table 20:** Improving Tasks and Costs for the Sales Process.

*PU*	*Sales Process*	*Cost*
***Task***		
***1***	***Remind suppliers about bill of lading***	***100***
*2*	*Issue Commercial order to foreign suppliers*	***400***
*3*	*Customs agent contacts the sales dept*. *about the new shipment*	***50***
*4*	*Bill of lading for the new shipment arrives at the sales dept*.	***100***
*5*	*Sales dept*. *receives the shipment at the warehouse*	***3000***
*6*	*Sales dept*. *enters the new shipment into the commercial database*	***300***
*7*	*Customers are contacted about the new shipment*	***1600***
***8***	*Money is deposited by the customers to the sales account*	***1000***
*9*	***Hire a mediator to confirm all the slips at the bank***	***250***
*10*	***Internet banking***	***3000***
*11*	*Invoice is issued*	***1000***
*12*	*Seed is sent to the customers and deducted from the Commercial database*	*13000*
*13*	*Hire a sales agent to re-confirm paper recordings*	*270*
*14*	*All customers’ accounts are also recorded manually on paper*	*1000*

**Table 21:** Improving Tasks and Costs for the Customer management.

*PU*	*Customer management*	*Cost*
***Task***		
***1***	*The contact information are obtained by the Sales or Technical Dept*.	*250*
*2*	*The contact information is entered in the local copy of the Customers’ Database*	*250*
	***Instant correction of out of date information***	*40*
*3*	*The two copies of the Customers’ Database are synchronized*.	*225*
*4*	***Visiting technical team checks the customer information***	***50***

#### 4.3.2. Execution

***Execute the modified PU’s:***

In the Company it was determined that three months was enough time for the improving tasks to take effect. Hence, the PU’s were executed over a three-month period. Note that during this time the business of the Company was not hampered at all, and the authors were only monitoring the effects of the newly-designed processes as the Company was pushing ahead with its business objectives. Also depending on the size and the nature of the organization the three-month period may vary.

***Analysis of the Execution:***

After the execution period DQPi’s were measured again to determine possible improvements. The improvements were used to calculate awards for the improving tasks. The new improvements, awards, and DuQ values after execution 1 are shown in Tables 22, 23 and 24 respectively.

**Table 22:** DQP Measurements after Execution 1.

***DU***	***DQP***	***Initial Measurement***	***Exec 1***	***Measurement 2***	***Improvement***
***Sample Database***	*Consistency of Grams/ Grain*	*0*.*5*		*0*.*45*	*-0*.*05*
***Sample Database***	*Sample Data Base Accuracy*	*0*.*8*		*0*.*9*	*0*.*1*
***Sample Database***	*Duplicate Names*	*0*.*9*		*0*.*85*	*-0*.*05*
***Commercial Database***	*Readable Bank Slip*	*0*.*5*		*1*	*0*.*5*
***Commercial Database***	*Accuracy of Paper Recordings of Sales*	*0*.*85*		*1*	*0*.*15*
***Commercial Database***	*Accuracy of Bill of Lading*	*0*.*8*		*0*.*83*	*0*.*03*
***Customers’ Database***	*Accuracy of Customers’ Database*	*0*.*8*		*0*.*9*	*0*.*1*
***Customers’ Database***	*Completeness of Customers’ Database*	*0*.*7*		*0*.*85*	*0*.*15*

**Table 23:** Awards after Execution 1.

Task	Process Unit		*Consistency of Grams/ Grain*	*Accuracy of Sample Database*	*Duplicate Names*	*Readable Bank Slip*	*Accuracy of Paper Recordings*	*Accuracy of Bill of Lading*	*Accuracy of Customers’ Database*	*Completeness of Customers’ Database*
***1***	*Technical*	*Remind suppliers about grams / grains*	*-4*.*16*							
**2**	*Technical*	*Corrective records*		*15*.*91*						
***3***	*Sales*	*Remind suppliers about bill of lading*						*3*.*53*		
**4**	*Sales*	*Hire a mediator to confirm all the slips at the bank*				*58*.*1*				
***5***	*Sales*	*Internet banking*				*73*.*06*				
**6**	*Sales*	*Hire a sales agent to re-confirm paper recordings*					*29*			
***7***	*Customer management*	*Instant correction of out of date information*							*5*.*82*	*9*.*41*
**8**	*Customer management*	*Visiting technical team checks the contact information*							*5*.*75*	*9*.*25*

**Table 24:** DuQ Values after Execution 1.

***DU***	***Initial DuQ***	***Exec 1***	***DuQ2***
***Sample Database***	*1*.*42*		*1*.*4735*
***Commercial Database***	*1*.*04*		*1*.*6039*
***Customers’ Database***	*1*.*15*		*1*.*325*

The analysis of improvement and cost shows that the improving task “*Remind suppliers about grams / grains*” was not effective at all, because not only improvement was not gained, but also the related DQPi worsened during the execution. We found the reason to be that foreign suppliers cannot abide by the Company requests regarding this issue. The same is almost true for the improving task “*Remind suppliers about bill of lading*” which produced minimal improvement. Notice, that the above two improving tasks were the only “notifications” that were designed. Since the PU’s that caused the respective DQPi’s were outside of Company (i.e. foreign suppliers’ PU’s), the only thing that could be done was to send a notifications.

The DQPi “*Readable Bank Slip*” had an excellent improvement to a perfect one. Out of the two improving tasks “Internet Banking” had a better award because of smaller cost.

For the Customers’ Database, the two DQPi’s had similar but modest improvements. The associated improving tasks also had similar costs.

### 4.4. Repeating the TBDQ Cycle for Company

Since the case study involves a small company for the next two repetitions of the TBDQ cycle the planning step did not vary significantly. For simplicity purposes only the most significant activities of the evaluation, evolution, and execution are discussed. The second and third executions were also done in three-month periods.

#### 4.4.1. Second Execution of the Company Case

In the second execution the Experts team largely focused on the DQPi’s with more than one improving task (i.e. Accuracy of Sample Data Base, Readable Bank Slip, Accuracy of Customers’ Database). In order to find out which improving tasks were more effective, they were executed one at a time. Also unsuccessful improving tasks were not included in this implementation. The above modifications caused the following changes:

1. *“Remind suppliers about grams / grains” was removed from the technical process*.
2. *“Remind suppliers about bill of lading” was removed from the sales process*.
3. *“Hire a bank mediator to confirm slips at the bank” was removed from the sales process*.
4. *“Visiting technical team checks the contact information” was removed from customer management*.

The new improvements, awards, and DuQ values after execution 2 are shown in Tables 25, 26 and 27 respectively.

**Table 25:** DQPi Measurements after Execution 2.

***DU***	***DQP***	***Initial Measurement***	***Exec 1***	***Measurement 2***	***Exec 2***	***Measurement 3***	***Improvement***
***Sample Database***	*Consistency of Grams/ Grain*	*0*.*5*		*0*.*45*		*0*.*46*	*0*.*01*
***Sample Database***	*Sample Data Base Accuracy*	*0*.*8*		*0*.*9*		*0*.*92*	*0*.*02*
***Sample Database***	*Duplicate Names*	*0*.*9*		*0*.*85*		*0*.*83*	*-0*.*02*
***Commercial Database***	*Readable Bank Slip*	*0*.*5*		*1*		*1*	*0*
***Commercial Database***	*Accuracy of Paper Recordings of Sales*	*0*.*85*		*1*		*0*.*98*	*-0*.*02*
***Commercial Database***	*Accuracy of Bill of Lading*	*0*.*8*		*0*.*83*		*0*.*83*	*0*
***Customers’ Database***	*Accuracy of Customers’ Database*	*0*.*8*		*0*.*9*		*0*.*91*	*0*.*1*
***Customers’ Database***	*Completeness of Customers’ Database*	*0*.*7*		*0*.*85*		*0*.*88*	*0*.*03*

**Table 26:** Awards after Execution 2.

*Task*	*Process Unit*		*Consistency of Grams/ Grain*	*Accuracy of Sample Database*	*Duplicate Names*	*Readable Bank Slip*	*Accuracy of Paper Recordings*	*Accuracy of Bill of Lading*	*Accuracy of Customers’ Database*	*Completeness of Customers’ Database*
***1***	*Technical*	*Corrective records*		*2*.*2*						
***2***	*Sales*	*Internet banking*				*0*				
***3***	*Sales*	*Hire a sales agent to re-confirm paper recordings*					*-1*.*84*			
***4***	*Customer management*	*Instant correction of out of date information*							*1*.*03*	*3*.*28*

**Table 27:** DuQ Values after Execution 2.

***DU***	***Initial DuQ***	***Exec 1***	***DuQ2***	***Exec 2***	***DuQ3***
***Sample Database***	*1*.*42*		*1*.*4735*		*1*.*4802*
***Commercial Database***	*1*.*04*		*1*.*6039*		*1*.*5973*
***Customers’ Database***	*1*.*15*		*1*.*325*		*1*.*5602*

For the DQPi “Consistency of grams / Grains”, although there was no improving task, the DQPi gained negligible improvement which is probably due to sheer chance.

The award for improving task “Corrective records” is small. However, since it is positive thru first and second execution, it shows that it slowly but consistently improves the accuracy of the sample database. If a rapid improvement is needed probably a more rigorous improving task is needed.

For improving task “Internet banking” the reward is zero. However, remember that the related DQPi was at 1 (i.e. best value) when the cycle started. This means that internet banking alone could maintain the DQPi “Readable bank slip”, even though another more expensive improving task was removed from the PU (i.e. Hire a bank mediator). Hence, it seems logical to leave out the expensive improving task.

As for the DQPi “Accuracy of Paper Recordings” the respective improving task had a negligible negative award, which means the improving task (i.e. Hire a sales agent to re-confirm paper recordings) can keep the DQPi at a respectable level (i.e. 0.98). The remaining issue is whether a less expensive improving task can be designed for the DQPi. However, no other improving task is designed yet.

For improving task “Instant correction of out date information” there are two negligible awards for the respective DQPi’s. Again, this means the task can maintain the related DQPi’s at the current levels (i.e. 0.88 and 0.91). If the current levels are acceptable no other task is needed. Remember that the accompanying task for the same related DQPi’s was “Visiting technical team checks the contact information” which was omitted after the first execution. The remaining question is whether the omitted improving task can produce a better reward

#### 4.4.2. Third Execution of the Company Case

In the third execution, it was decided that for the DQPi “Accuracy of the Sample Database” a value of 0.92 is not enough and other improving tasks must be used for it. Although the most natural suggestion seemed that a colleague or new employee double check the entries to the sample database, the DU owners (i.e. the technical team) rejected the idea and preferred that all data be entered by one person so that in case of errors the responsible party can be easily identified. The other suggested improving task was to perform a complete “Inventory Check” to bring the sample database up to an ideal accuracy. This task was inserted in the technical PU for execution 3. Also for the Customers’ Database the improving “Instant correction of out date information” was replaced by “Visiting technical team checks the contact information” to see which task is has a better reward.

The summary of the PU changes in execution 3 is as the following.

1. *“Inventory check” was added to the technical process*. *Cost = 250*
2. *“Visiting technical team checks the contact information” was added to customer management*.
3. *“Notification to Technical PU*: *Double-check new customers’ information” was added to the customer management*. *Cost = 40*
4. “Instant correction of out date information” was removed from customer management

The new improvements, awards, and DuQ values after execution 3 are shown in Tables 28, 29 and 30 respectively.

**Table 28:** DQPi Measurements after Execution 3.

***DU***	***DQP***	***Original Measurement***	***Exec 1***	***Measurement 2***	***Exec 2***	***Measurement 3***	***Exec 3***	***Measurement 4***	***Improvement***
***Sample Database***	*Consistency of Grams/ Grain*	*0*.*5*		*0*.*45*		*0*.*44*		*0*.*44*	*0*
***Sample Database***	*Sample Database Accuracy*	*0*.*8*		*0*.*9*		*0*.*92*		*1*	*0*.*08*
***Sample Database***	*Duplicate Names*	*0*.*9*		*0*.*85*		*0*.*83*		*0*.*83*	*0*
***Commercial Database***	*Readable Bank Slip*	*0*.*5*		*1*		*1*		*1*	*0*
***Commercial Database***	*Accuracy of Paper Recordings of Sales*	*0*.*85*		*1*		*0*.*98*		*1*	*0*.*02*
***Commercial Database***	*Accuracy of Bill of Lading*	*0*.*8*		*0*.*83*		*0*.*83*		*0*.*8*	*-0*.*03*
***Customers’ Database***	*Accuracy of Customers’ Database*	*0*.*8*		*0*.*9*		*0*.*91*		*0*.*84*	*-0*.*07*
***Customers’ Database***	*Completeness of Customers’ Database*	*0*.*7*		*0*.*85*		*0*.*88*		*0*.*88*	*0*

**Table 29:** Awards for Execution 3.

*Task*	*Process Unit*		*Consistency of Grams/ Grain*	*Accuracy of Sample Database*	*Duplicate Names*	*Readable Bank Slip*	*Accuracy of Paper Recordings*	*Accuracy of Bill of Lading*	*Accuracy of Customers’ Database*	*Completeness of Customers’ Database*
***1***	*Technical*	*Inventory check*		*4*.*37*						
***2***	*Technical*	*Corrective records*		*4*.*84*						
***3***	*Sales*	*Internet banking*				*0*				
***4***	*Sales*	*Hire a sales agent to re-confirm paper recordings*					*2*.*19*			
***5***	*Customer management*	*Visiting technical team checks the contact information*							*-3*.*97*	*0*
***6***	*Customer management*	*Notification to Technical PU*: *Double-check new customers’ information*							*-3*.*93*	*0*

**Table 30:** DuQ Values after Execution 3.

***DU***	***Initial DuQ***	***Exec 1***	***DuQ2***	***Exec 2***	***DuQ3***	***Exec 3***	***DuQ4***
***Sample Database***	*1*.*42*		*1*.*47*		*1*.*48*		*1*.*56*
***Commercial Database***	*1*.*04*		*1*.*60*		*1*.*60*		*1*.*59*
***Customers’ Database***	*1*.*15*		*1*.*33*		*1*.*56*		*1*.*28*

In this execution the improving task “inventory check” brought the sample database accuracy right up to an ideal value of one. Notice that the model assigns very close awards to the two tasks “inventory check” and “corrective records” (i.e. 9.55 and 11.69 respectively), even though the cost of “inventory check” is much larger. This is because at the beginning of the execution the respective DQPi did not have much room for improvement in the first place (i.e. 0.92). This makes the case that employing expensive tasks for DQPi’s with a high value may not be very cost effective. Also the improving task “Visiting technical team checks the contact information” produced a negative award due to negative improvement. This because the “frequency” of technical team visits to farms (10 every three months) is not enough to provide adequate feedback for the Customers’ Database. Since the Customers’ Database is losing about 5% accuracy a year a more frequent improving task is needed. Lastly, “*Notification to Technical PU*: *Double-check new customers’ information”* did not produce any significant improvement. This was probably because the technical team seldom makes mistakes recording the contact information of the new customers. Hence, sending them a notification did not have significant improvement.

**Fig 13:**
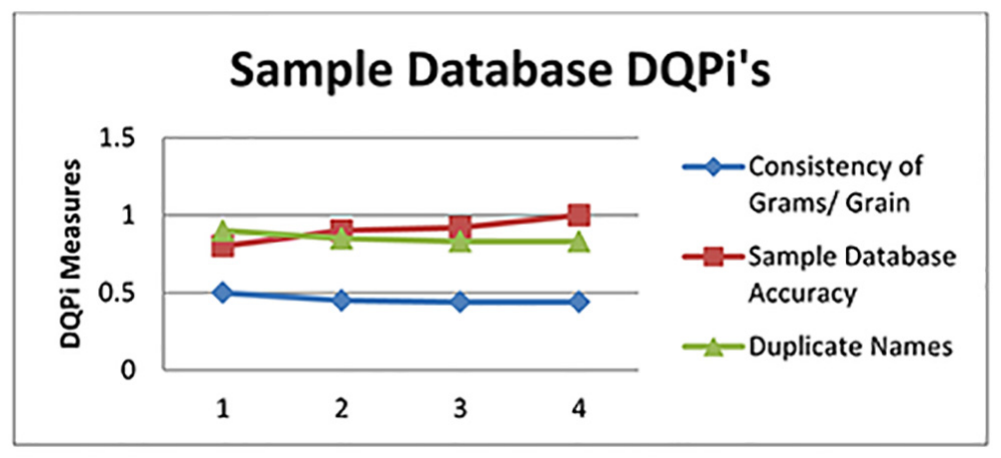
DQPi’s for the Sample Database.

**Fig 14:**
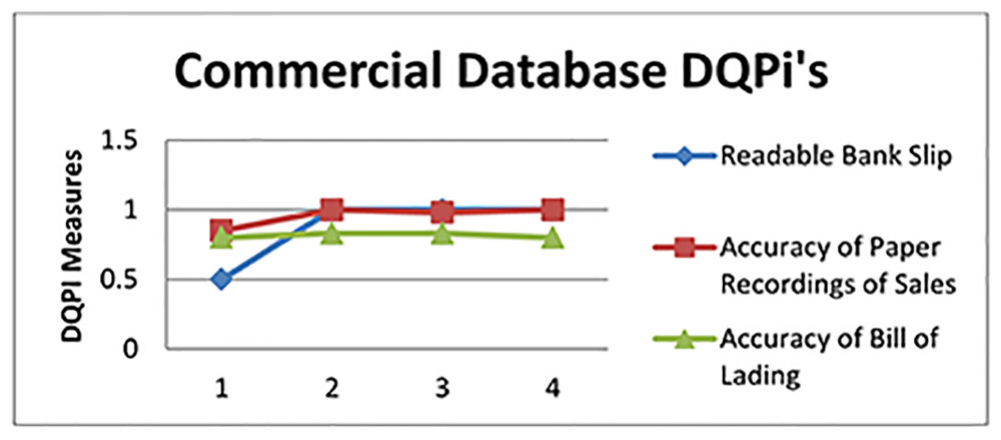
DQPi’s for the Commercial Database.

**Fig 15:**
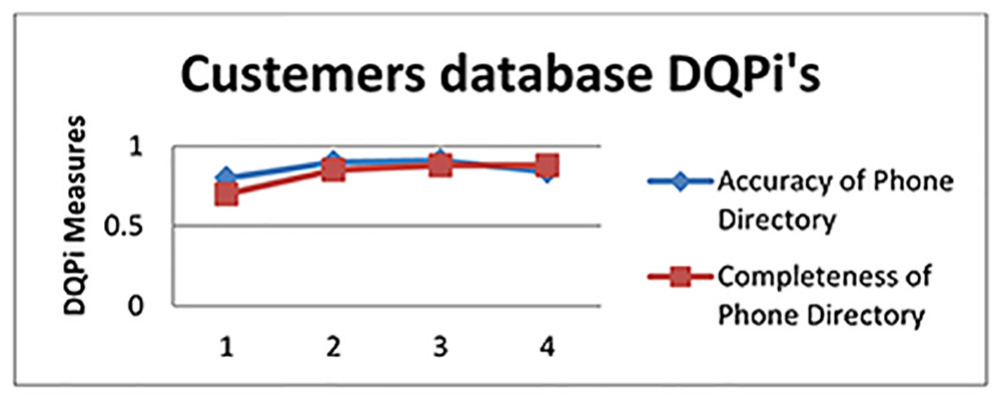
DQPi’s for the Customers’ Database.

The DQPi values after the executions are illustrated for the three DU’s in Figs 13, 14 and 15.

**Fig 16:**
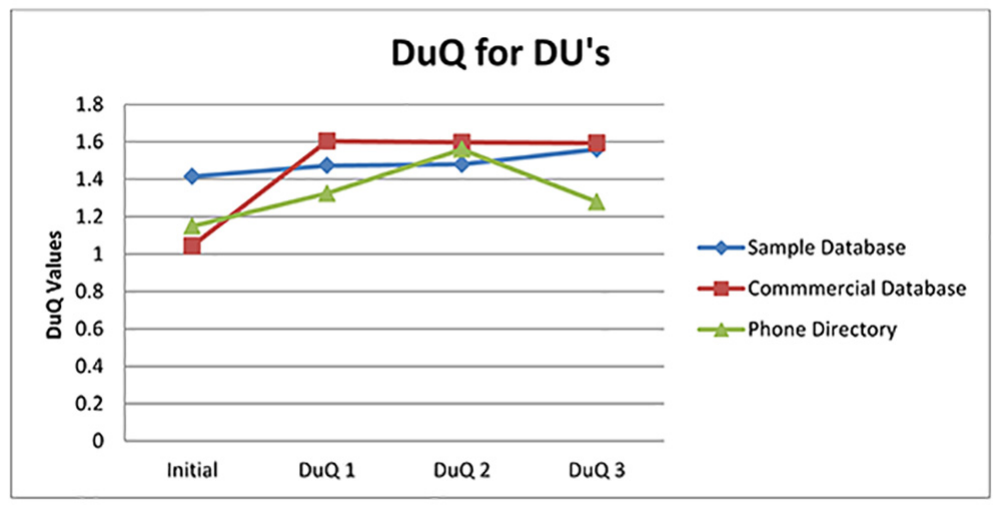
DuQ Values.

Fig 16 shows DU qualities change after each execution.

## 5. Discussion and Conclusion

In this paper a new DQ method was introduced which continuously modifies the process units of an organization to improve DQ. Modifying the processes involves inserting new improving tasks into them in order to better counter the effects of risky tasks.

The effects of the improving tasks are measured by an award system which is based on the improving task *cost* and the *improvement* achieved on DQ problems. Some of the prominent issues about the method that were discovered thru the Behta case study are the following.

### Subjective Survey

It remains to be studied how the subject surveys in a DQ method could produce more accurate and more consistent results. One solution could be to educate the participating subjects about DQ so that they will answer the survey questionnaires with better knowledge. Also, as proposed in [7], it may be practical to include several "items" for each dimension in the questionnaire in order to measure a dimension from different angles and views.

### DQ Budget

One of the improving tasks designed (i.e. Purchase DB Application) was never used because of high cost. This happened mainly because no clear budget was defined for DQ. At the absence of such a budget the Experts cannot determine which of the tasks will actually be performed and the final decision is left to the higher level management.

### Outside Dependency

If an improving task is somehow dependent on outside help it cannot be very relied upon. In the case study two tasks that notified the foreign suppliers about *grams/grains* and *bills of lading* did not gain an improvement at all because they relied heavily on outside cooperation. Improving tasks must be designed in a way to largely depend on assistance only from inside the organization.

### Multiple Improving Tasks for a DQPi

If several improving tasks are proposed for a single DQPi there are two approaches to using them. First, if the related DQPi has high significance all improving tasks may be utilized together to gain the maximum improvement in shortest possible time. Then the tasks could be used one by one in order to discover which has more improving or maintenance effect on the DQPi. On the other hand, if the DQPi is not very significant the improving tasks may be used one at a time in each execution to see which one has a better effect.

### Semi-structured and Unstructured data

TBDQ at its current form only assesses and improves structured data. As part of the future work the method could be extended to include semi-structured and unstructured data as well.

### Business Process Re-engineering

The proposed method only modified the organizational PU’s by adding new tasks to them. However, what happens if a PU’s must be radically re-designed to achieve better DQ. This is generally called *business process re-engineering* [5]. For instance, in our case study even though the technical and the commercial departments are in two buildings, they share the same phone directory. Since each department keeps its own local copy, contact information is entered independently and the local copies are periodically synchronized. One might argue that the process itself must be re-designed, because from a technical point of view it is better to have a single copy on a common *server*, with proper access controls, rather than two independent copies. This required the re-design of *contact management* PU in Behta as well as procurement of new equipment. This met resistance in the case study because of the cost and security concerns. In fact, business process re-engineering is often very expensive, and hence, is likely to meet resistance.

As future work, one of the remaining issues is human error factor. In TBDQ most tasks were performed by human agents, and human operations usually involve an error margin. Hence, even if improving tasks are well-designed human error might hamper their improving ability. Therefore, the human error factor must be incorporated in the future work of the TBDQ.

Another issue is to determine if business process re-engineering is a good choice for an organization. What are the operational details and how is the heavy cost justified. Finally how can a process-driven method, like TBDQ, be extended to a method that re-designs entire organizational processes?

## Supporting Information

S1 Text Signed Behta Consent From for Data Quality Research. (JPEG)

S2 Text Research Questionnaire for Significance of Dimensions. (DOCX)
