# Supplementary figures and images for: TBDQ: A Pragmatic Task-Based Method to Data Quality Assessment and Improvement
Source: PLoS One. 2016 May 18;11(5):e0154508. doi: 10.1371/journal.pone.0154508 (PMC4871700; doi:10.1371/journal.pone.0154508)

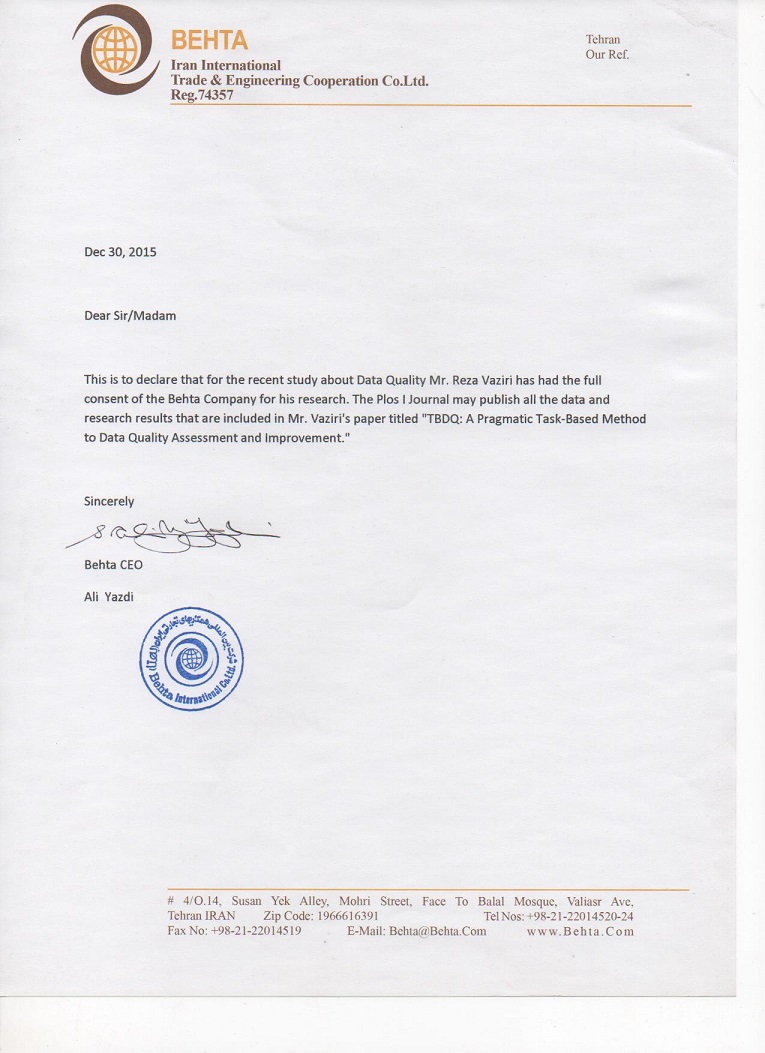

Supplement: S1 Text — (JPEG) [file pone.0154508.s001.jpeg]
