# Supplementary material for: TBDQ: A Pragmatic Task-Based Method to Data Quality Assessment and Improvement
Source: PLoS One. 2016 May 18;11(5):e0154508. doi: 10.1371/journal.pone.0154508 (PMC4871700; doi:10.1371/journal.pone.0154508)
Supplement: S2 Text — (DOCX) [file pone.0154508.s002.docx]

First Name: Last Name:

Position: Department:

Dear Colleague

Please indicate how "significant" each of the following dimensions are for the Behta Company?

**Accuracy**: extent to which data come close to the actual value.

Not all Medium Extremely

0 1 2 3 4 5 6 7 8 9 10

**Accessibility**: extent to which data is available, or easily and quickly retrievable.

Not all Medium Extremely

0 1 2 3 4 5 6 7 8 9 10

**Appropriate Amount**: extent to which the volume of data is appropriate for the task at hand.

Not all Medium Extremely

0 1 2 3 4 5 6 7 8 9 10

**Believability**: extent to which data is regarded as true and credible.

Not all Medium Extremely

0 1 2 3 4 5 6 7 8 9 10

**Completeness**: extent to which data is not missing and has sufficient breadth and depth for the task at hand.

Not all Medium Extremely

0 1 2 3 4 5 6 7 8 9 10

**Understandability**: extent to which data is easily comprehended.

Not all Medium Extremely

0 1 2 3 4 5 6 7 8 9 10

**Value-Added**: extent to which data is beneficial and provides advantages from its use.

Not all Medium Extremely

0 1 2 3 4 5 6 7 8 9 10

**Relevancy**: extent to which data is applicable and helpful for the task at hand.

Not all Medium Extremely

0 1 2 3 4 5 6 7 8 9 10

**Reputation**: extent to which data is highly regarded in terms of its source or content.

Not all Medium Extremely

0 1 2 3 4 5 6 7 8 9 10

**Security**: extent to which data is restricted appropriately to maintain its security.

Not all Medium Extremely

0 1 2 3 4 5 6 7 8 9 10

**Timeliness**: extent to which data is sufficiently up-to-date for the task at hand.

Not all Medium Extremely

0 1 2 3 4 5 6 7 8 9 10

**Free-of-Error**: extent to which data is correct and reliable.

Not all Medium Extremely

0 1 2 3 4 5 6 7 8 9 10

**Interpretability**: extent to which data is in appropriate languages, symbols, and units and the definitions are clear.

Not all Medium Extremely

0 1 2 3 4 5 6 7 8 9 10

**Objectivity**: extent to which data is unbiased, unprejudiced, and impartial.

Not all Medium Extremely

0 1 2 3 4 5 6 7 8 9 10

**References**

[1] Pipino, L., Lee, Y., & Wang, R. (2002). Data quality assessment. *Communication of ACM, 45*(4), 211-218.
